# The impact of body weight and diabetes on new-onset atrial fibrillation: a nationwide population based study

**DOI:** 10.1186/s12933-019-0932-z

**Published:** 2019-10-01

**Authors:** Yun Gi Kim, Kyung-Do Han, Jong-Il Choi, Ki Yung Boo, Do Young Kim, Suk-Kyu Oh, Kwang-No Lee, Jaemin Shim, Jin Seok Kim, Young-Hoon Kim

**Affiliations:** 1 Division of Cardiology, Korea University College of Medicine and Korea University Anam Hospital, Seoul, Republic of Korea; 2 Department of Biostatistics, College of Medicine, The Catholic University of Korea, Seoul, Republic of Korea; 3 Division of Cardiology, Department of Internal Medicine, Korea University College of Medicine and Korea University Medical Center, 73 Inchon-ro, Seongbuk-gu, Seoul, 02841 Republic of Korea

**Keywords:** Atrial fibrillation, Body weight, Waist circumference, Diabetes

## Abstract

**Background:**

Being obese or underweight, and having diabetes are important risk factors for new-onset atrial fibrillation (AF). However, it is unclear whether there is any interaction between body weight and diabetes in regard to development of new-onset AF. We aimed to evaluate the role of body weight status and various stage of diabetes on new-onset AF.

**Methods:**

This was a nationwide population based study using National Health Insurance Service (NHIS) data. A total of 9,797,418 patients who underwent national health check-ups were analyzed. Patients were classified as underweight [body mass index (BMI) < 18.5], normal reference group (18.5 ≤ BMI < 23.0), upper normal (23.0 ≤ BMI < 25.0), overweight (25.0 ≤ BMI < 30.0), or obese (BMI ≥ 30.0) based on BMI. Diabetes were categorized as non-diabetic, impaired fasting glucose (IFG), new-onset diabetes, diabetes < 5 years, and diabetes ≥ 5 years. Primary outcome end point was new-onset AF. New-onset AF was defined as one inpatient or two outpatient records of International Classification of Disease, Tenth Revision (ICD-10) codes in patients without prior AF diagnosis.

**Results:**

During 80,130,161 patient*years follow-up, a total of 196,136 new-onset AF occurred. Obese [hazard ration (HR) = 1.327], overweight (HR = 1.123), upper normal (HR = 1.040), and underweight (HR = 1.055) patients showed significantly increased risk of new-onset AF compared to the normal reference group. Gradual escalation in the risk of new-onset AF was observed along with advancing diabetic stage. Body weight status and diabetes were independently associated with new-onset AF and at the same time, had synergistic effects on the risk of new-onset AF with obese diabetic patients having the highest risk (HR = 1.823).

**Conclusions:**

Patients with obesity, overweight, underweight, and diabetes had significantly increased risk of new-onset AF. Body weight status and diabetes had synergistic effects on the risk of new-onset AF. The risk of new-onset AF increased gradually with advancing diabetic stage. This study suggests that maintaining optimal body weight and glucose homeostasis might prevent new-onset AF.

## Background

Atrial fibrillation (AF) is the most common tachyarrhythmia and its incidence is estimated to increase substantially due to the aging of general population [1–3]. Quality of life is significantly decreased in patients with AF [1, 4, 5]. Furthermore, the risk of stroke, heart failure, and death is significantly increased when AF is present [6–8]. Radiofrequency catheter ablation (RFCA) is highly effective in managing AF. Its efficacy in preventing AF recurrence is shown by improved quality of life, reduced stroke risk, preserved heart function, and even reduced all-cause mortality in a subgroup of patients [1, 9–11]. However, probably due to the high efficacy of RFCA, the upstream therapy which is the primary prevention for AF, did not receive sufficient interest. In order to prevent AF, the exact etiology of AF should be understood. Currently identified risk factors for AF include advanced age, hypertension, heart failure, ischemic heart disease, and various inflammatory diseases [7, 12]. Diabetes, a powerful risk factor for ischemic heart disease, is also known to increase the risk of new-onset AF [12–14]. Obesity is another risk factor for AF [15, 16]. Previous studies demonstrated that one kg/m^2^ increase in body mass index (BMI) is correlated with a 3–5% increase in the risk of new-onset AF [17–19]. The underlying mechanism of such an association between new-onset AF and diabetes/obesity is not clear but it is proposed that increased left atrial size and pressure might contribute to the development of AF in these patients [20].

Obesity is associated with an increased risk of diabetes and diabetic patients are likely to become obese due to insulin resistance [21]. It is unclear whether there is any interaction between diabetes and obesity in regard to development of new-onset AF. Interestingly, being underweight is also associated with new-onset AF independent of confounding factors such as chronic lung disease and malignancy [17]. The association between being underweight and new-onset AF complicates the elucidation of the true relationship between diabetes, body weight, and new-onset AF. The aim of the current study was to identify the precise impact of body weight and diabetes on the development of AF through various subgroup analyses.

## Methods

### Patients

The National Health Insurance Service (NHIS) managed by the government is the single medical insurer in the Republic of Korea. The majority of Korean people (97.1%) are mandatory subscribers and the database is open to medical researchers whose study protocols are approved by the official review committee (https://nhiss.nhis.or.kr/). The remaining 3% of the population are medical aid persons. The database contains demographics, diagnosis codes, use of inpatient and outpatient services, pharmacy dispensing claims, and mortality data. The K-NHIS offers a regular national health check-up for all subscribers and it includes health questionnaires, laboratory tests including fasting blood sugar and chest X-ray, and measurement of body weight, height, and waist circumference. The current study included patients who underwent a national health check-up in 2009. The authors have no conflict of interest with NHIS. This study was approved by the Institutional Review Board of Korea University Medical Center Anam Hospital and informed consent was waived.

### Definitions

The diagnosis of AF required one inpatient or two outpatient records of International Classification of Disease, Tenth Revision (ICD-10) codes in the database. The exact diagnosis code for AF is presented in Additional file 1: Table S1.

Body weight status was classified into five groups according to BMI: underweight [BMI < 18.5 (kg/m^2^)], normal reference (18.5 ≤ BMI < 23.0), upper normal (23.0 ≤ BMI < 25.0), overweight (25.0 ≤ BMI < 30.0), and obesity (BMI ≥ 30.0). Waist circumference (WC) was also utilized to complement BMI and was classified into 6 stages: (i) WC < 80.0 (cm), 80.0 ≤ WC < 85.0, 85.0 ≤ WC < 90.0, 90.0 ≤ WC < 95.0, 95.0 ≤ WC < 100.0, and WC ≥ 100.0 for males and (ii) WC < 75.0, 75.0 ≤ WC < 80.0, 80.0 ≤ WC < 85.0, 85.0 ≤ WC < 90.0, 90.0 ≤ WC < 95.0, and WC ≥ 95.0 for females.

Only type 2 diabetes was included in this study. Patients were classified into 5 stages with regard to diabetes: non-diabetic, impaired fasting glucose (IFG), new-onset diabetes, diabetic for less than 5 years, and diabetic for more than 5 years. Each stage was defined as follows: (i) non-diabetic as fasting blood sugar (FBS) < 100 mg/dl, no use of diabetic medication and no history of physician-diagnosed diabetes, (ii) IFS: FBS 100–125 mg/dl, no use of diabetic medication and no history of physician-diagnosed diabetes, (iii) diabetic: FBS ≥ 126 mg/dl or use of diabetic medication or history of physician-diagnosed diabetes. New-onset diabetes was defined as those diagnosed with diabetes at the time of National health check-ups.

### Study end points

The occurrence of new-onset AF was the end point of this study. Incidence of new-onset AF was defined as number of new-onset AF calculated for 1000 patient*years follow up. The influence of body weight status and different stage of diabetes were evaluated through various subgroup analyses.

### Statistical analysis

Continuous variables are described as mean ± standard deviation, and were compared using the Student’s t-test. Categorical variables are presented as percentile values, and were compared with a chi-square test or Fisher’s exact test as appropriate. Cox regression analysis was performed to calculate the hazard ratio (HR) and its 95% confidence interval (CI). Both univariate and multivariate analysis was performed through Cox regression analysis. Model 1 was adjusted for age and sex. Model 2 was adjusted for age, sex, smoking status, alcohol consumption status, physical activity, income level, diabetes, hypertension, and dyslipidemia. All significance tests were two-tailed, and p values equal or less than 0.05 were considered statistically significant. All statistical analyses were performed with SAS version 9.2 (SAS Institute, Cary, NC).

## Results

### Patients

**Fig. 1:**
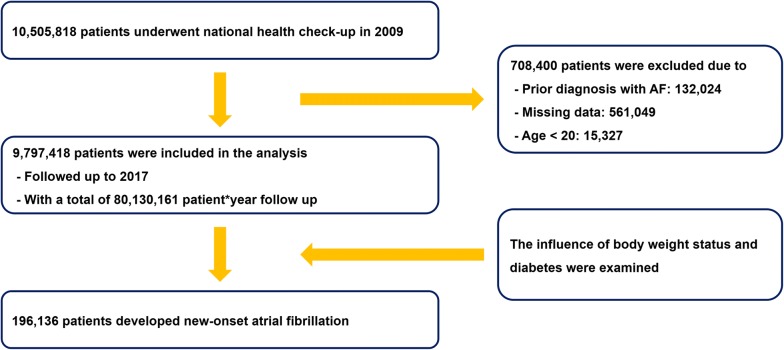
Study flow. *K-NHIS* Korean National Health Insurance Service

A total of 9,797,418 patients were included in the analysis. Baseline characteristics of the study population are summarized in Table 1. A total of 842,848 (9.41%) patients were diagnosed with diabetes: 287,417; 289,883; and 265,548 patients as newly diagnosed, less than 5 years, and over 5 years diabetes, respectively. A flowchart of the study population enrollment is presented in Fig. 1. Baseline demographics of patients with and without diabetes are described in Table 1. In brief, patients with diabetes showed a higher percentage of male sex, older age, higher BMI, and higher prevalence of hypertension and dyslipidemia.

**Table 1:** Baseline demographic of patients with and without diabetes

	Total	Non-diabetic	Diabetic	p value
	(N = 9,797,418)	(n = 8,954,570)	(n = 842,848)	
Male	5,356,943 (54.68%)	4,838,750 (54.04%)	518,193 (61.48%)	< 0.0001
Smoking				< 0.0001
Non-smoker	5,832,072 (59.53%)	5,363,620 (59.90%)	468,452 (55.58%)	
Ex-smoker	1,394,168 (14.23%)	1,240,121 (13.85%)	154,047 (18.28%)	
Current-smoker	2,571,178 (26.24%)	2,350,829 (26.25%)	220,349 (26.14%)	
Alcohol consumption				< 0.0001
Non-drinker	5,037,180 (51.41%)	4,558,687 (50.91%)	478,493 (56.77%)	
Mild-drinker	4,087,517 (41.72%)	3,796,029 (42.39%)	291,488 (34.58%)	
Heavy-drinker	672,721 (6.87%)	599,854 (6.70%)	72,867 (8.65%)	
Regular exercise (yes)	5,022,112 (51.26%)	4,608,738 (51.47%)	413,374 (49.04%)	< 0.0001
Income (lower quartile)	2,590,673 (26.44%)	2,361,426 (26.37%)	229,247 (27.20%)	< 0.0001
Hypertension	2,486,705 (25.38%)	2,007,127 (22.41%)	479,578 (56.90%)	< 0.0001
Dyslipidemia	1,773,560 (18.10%)	1,429,021 (15.96%)	344,539 (40.88%)	< 0.0001
Diabetes 5 level				< 0.0001
Non-diabetic	6,739,088 (68.78%)	6,739,088 (75.26%)		
IFG	2,215,482 (22.61%)	2,215,482 (24.74%)		
New-onset diabetes	287,417 (2.93%)		287,417 (34.10%)	
Diabetes < 5 years	289,883 (2.96%)		289,883 (34.39%)	
Diabetes ≥ 5 years	265,548 (2.71%)		265,548 (31.51%)	
Age (years)	47.02 ± 14.06	46.05 ± 13.85	57.25 ± 12.03	< 0.0001
Height (cm)	163.89 ± 9.22	164.03 ± 9.21	162.46 ± 9.17	< 0.0001
Weight (kg)	63.90 ± 11.59	63.69 ± 11.57	66.22 ± 11.50	< 0.0001
Body mass index (kg/m^2^)	23.70 ± 3.21	23.57 ± 3.17	25.01 ± 3.27	< 0.0001
Waist circumference (cm)	80.19 ± 9.07	79.69 ± 8.97	85.45 ± 8.38	< 0.0001
Systolic blood pressure (mmHg)	122.38 ± 14.94	121.75 ± 14.71	129.13 ± 15.69	< 0.0001
Diastolic blood pressure (mmHg)	76.28 ± 9.97	76.02 ± 9.92	79.10 ± 10.12	< 0.0001
Fasting glucose (mg/dl)	97.07 ± 22.85	92.50 ± 11.50	145.63 ± 45.66	< 0.0001
Total cholesterol (mg/dl)	195.07 ± 36.59	194.86 ± 36.07	197.33 ± 41.60	< 0.0001
High density lipoprotein (mg/dl)	55.43 ± 18.86	55.77 ± 18.64	51.79 ± 20.73	< 0.0001

### BMI and new-onset AF

During the mean follow-up duration of 8.18 ± 1.01 years (80,130,161 patient*years follow-up), a total of 196,136 new-onset AF were diagnosed. Patients in the higher BMI group showed a higher incidence of new-onset AF. After multivariate adjustment, obese patients showed significantly higher risk for new-onset AF compared to the normal reference group (HR = 1.327; 95% CI 1.298–1.257; p < 0.001; Table 2). Overweight patients also had an increased risk of new-onset AF (HR = 1.123; 95% CI 1.111–1.136; p < 0.001; Table 2) but to a lesser degree compared with obese patients. Patients with upper normal BMI showed a minimally increased risk (HR = 1.040; 95% CI 1.028–1.052; p < 0.001; Table 2) compared with the normal reference group. The influence of BMI on the risk of new-onset AF was maintained in both non-diabetic and diabetic patients with overweight and obese patients showing a significantly increased risk of new-onset AF (Table 2). Patients with upper normal BMI showed a modest increase in the risk of new-onset AF in the non-diabetic group (HR = 1.050; 95% CI 1.036–1.063; p < 0.001; Table 2) and a similar risk was seen in the diabetic group (HR = 0.970; 95% CI 0.940–1.000; p = 0.050; Table 2). Patients who were underweight also showed a higher incidence of new-onset AF (HR = 1.055; 95% CI 1.026–1.085; p < 0.001; Table 2). Being underweight was a significant risk factor for development of new-onset AF in both non-diabetic (HR = 1.056; 95% CI 1.025–1.087; p < 0.001; Table 2) and diabetic groups (HR = 1.119; 95% CI 1.024–1.224; p < 0.001; Table 2). However, if patients with IFG were classified as diabetic, underweight patients without diabetes and IFG did not show an increased risk of new-onset AF (HR = 1.008; 95% CI 0.975–1.042; Additional file 1: Table S2). The impact of BMI on new-onset AF was maintained throughout different diabetes stages with obese patients having the highest risk of developing AF (Additional file 1: Table S2).

**Table 2:** Impact of body weight status and diabetes on new-onset atrial fibrillation

	n	Event	Patient*years	Incidence	Model 1 (95% CI)	Model 2 (95% CI)	Model 3 (95% CI)
Total							
BMI							
< 18.5	374,501	5565	3,019,377	1.843	1.020 (0.993–1.049)	1.055 (1.026–1.085)	1.053 (1.025–1.083)
18.5–23	3,867,084	62,946	31,641,170	1.989	1 (reference)	1 (reference)	1 (reference)
23–25	2,423,219	50,163	19,850,785	2.527	1.125 (1.112–1.138)	1.040 (1.028–1.052)	1.042 (1.030–1.055)
25–30	2,795,090	68,242	22,864,770	2.985	1.290 (1.276–1.304)	1.123 (1.111–1.136)	1.128 (1.115–1.140)
30–	337,524	9220	2,754,059	3.348	1.606 (1.572–1.642)	1.327 (1.298–1.357)	1.325 (1.296–1.354)
WC (M/F)							
< 80/75	3,621,432	44,263	29,751,411	1.488	0.726 (0.717–0.736)	0.829 (0.818–0.840)	0.836 (0.825–0.848)
− 85/80	2,327,214	43,245	19,058,845	2.269	0.870 (0.859–0.882)	0.921 (0.909–0.934)	0.927 (0.915–0.939)
− 90/85	1,936,668	46,009	15,812,732	2.910	1 (reference)	1 (reference)	1 (reference)
− 95/90	1,134,028	33,661	9,219,448	3.651	1.148 (1.132–1.164)	1.096 (1.080–1.111)	1.092 (1.076–1.107)
− 100/95	504,432	17,713	4,085,267	4.336	1.252 (1.230–1.274)	1.176 (1.155–1.196)	1.162 (1.142–1.183)
≥ 100/95	273,644	11,245	2,202,458	5.106	1.615 (1.582–1.649)	1.410 (1.381–1.440)	1.369 (1.341–1.398)
Non-diabetic + IFG							
BMI							
< 18.5	361,941	5055	2,931,149	1.725	1.034 (1.004–1.064)	1.056 (1.025–1.087)	1.058 (1.028–1.089)
18.5–23	3,653,082	54,833	29,975,508	1.829	1 (reference)	1 (reference)	1 (reference)
23–25	2,206,558	41,998	18,127,691	2.317	1.129 (1.114–1.143)	1.050 (1.036–1.063)	1.049 (1.036–1.062)
25–30	2,453,625	54,545	20,131,059	2.709	1.288 (1.273–1.303)	1.134 (1.120–1.148)	1.133 (1.119–1.147)
30–	279,364	6663	2,287,495	2.913	1.541 (1.502–1.581)	1.309 (1.275–1.343)	1.294 (1.261–1.327)
WC (M/F)							
< 80/75	3,490,482	40,229	28,724,841	1.401	0.727 (0.717–0.737)	0.820 (0.808–0.832)	0.833 (0.821–0.845)
− 85/80	2,148,892	37,280	17,642,987	2.113	0.872 (0.859–0.884)	0.918 (0.905–0.931)	0.926 (0.913–0.940)
− 90/85	1,722,709	37,924	14,109,867	2.688	1 (reference)	1 (reference)	1 (reference)
− 95/90	968,520	26,661	7,904,775	3.373	1.154 (1.136–1.172)	1.107 (1.089–1.124)	1.101 (1.083–1.118)
− 100/95	412,804	13,266	3,359,409	3.949	1.244 (1.219–1.269)	1.185 (1.161–1.208)	1.163 (1.140–1.186)
≥ 100/95	211,163	7734	1,711,022	4.520	1.594 (1.556 –1.634)	1.418 (1.384–1.454)	1.365 (1.332–1.399)
Diabetic							
BMI							
< 18.5	12,560	510	88,228	5.780	1.096 (1.002–1.198)	1.119 (1.024–1.224)	1.100 (1.006–1.203)
18.5–23	214,002	8113	1,665,662	4.871	1 (reference)	1 (reference)	1 (reference)
23–25	216,661	8165	1,723,094	4.739	0.993 (0.963–1.024)	0.970 (0.940–1.000)	0.972 (0.942–1.002)
25–30	341,465	13,697	2,733,711	5.010	1.104 (1.074–1.134)	1.053 (1.024–1.082)	1.054 (1.025–1.084)
30–	58,160	2557	466,564	5.480	1.486 (1.421–1.554)	1.362 (1.301–1.425)	1.341 (1.282–1.403)
WC (M/F)							
< 80/75	130,950	4034	1,026,570	3.930	0.920 (0.886–0.955)	0.963 (0.927–1.001)	0.965 (0.929–1.003)
− 85/80	178,322	5965	1,415,858	4.213	0.925 (0.895–0.957)	0.945 (0.914–0.977)	0.949 (0.917–0.981)
− 90/85	213,959	8085	1,702,865	4.748	1 (reference)	1 (reference)	1 (reference)
− 95/90	165,508	7000	1,314,673	5.325	1.084 (1.050–1.119)	1.062 (1.029–1.097)	1.054 (1.021–1.089)
− 100/95	91,628	4447	725,857	6.127	1.187 (1.144–1.232)	1.156 (1.114–1.200)	1.150 (1.108–1.193)
≥ 100/95	62,481	3511	491,435	7.144	1.503 (1.445–1.565)	1.419 (1.363–1.477)	1.371 (1.317–1.427)

Model 1: adjusted for age and sex

Model 2: adjusted for model 1 plus smoking, alcohol consumption, regular physical activity, social income, diabetes, hypertension, and dyslipidemia

Model 3: adjusted for model 2 plus end stage renal failure, history of heart failure or coronary artery disease, hyperthyroidism, malignancy, and chronic obstructive pulmonary disease

*BMI* body mass index, *CI* confidence interval, *F* female, *IFG* impaired fasting glucose, *M* male, *WC* waist circumference

### Waist circumference

Waist circumference was associated with new-onset AF. Patients with a waist circumference 90–100 for men and 85–95 for women showed a modest increase in the risk of developing new-onset AF (HR = 1.176; 95% CI 1.155–1.196; p < 0.001; Table 2) regardless of diabetes stage (Table 2). Patients with waist circumference ≥ 100 for men and ≥ 95 for women had a significantly increased risk of new-onset AF (HR = 1.410; 95% CI 1.381–1.440; p < 0.001; Table 2) independent of diabetes stage (Table 2).

### Diabetes and new-onset AF

**Fig. 2:**
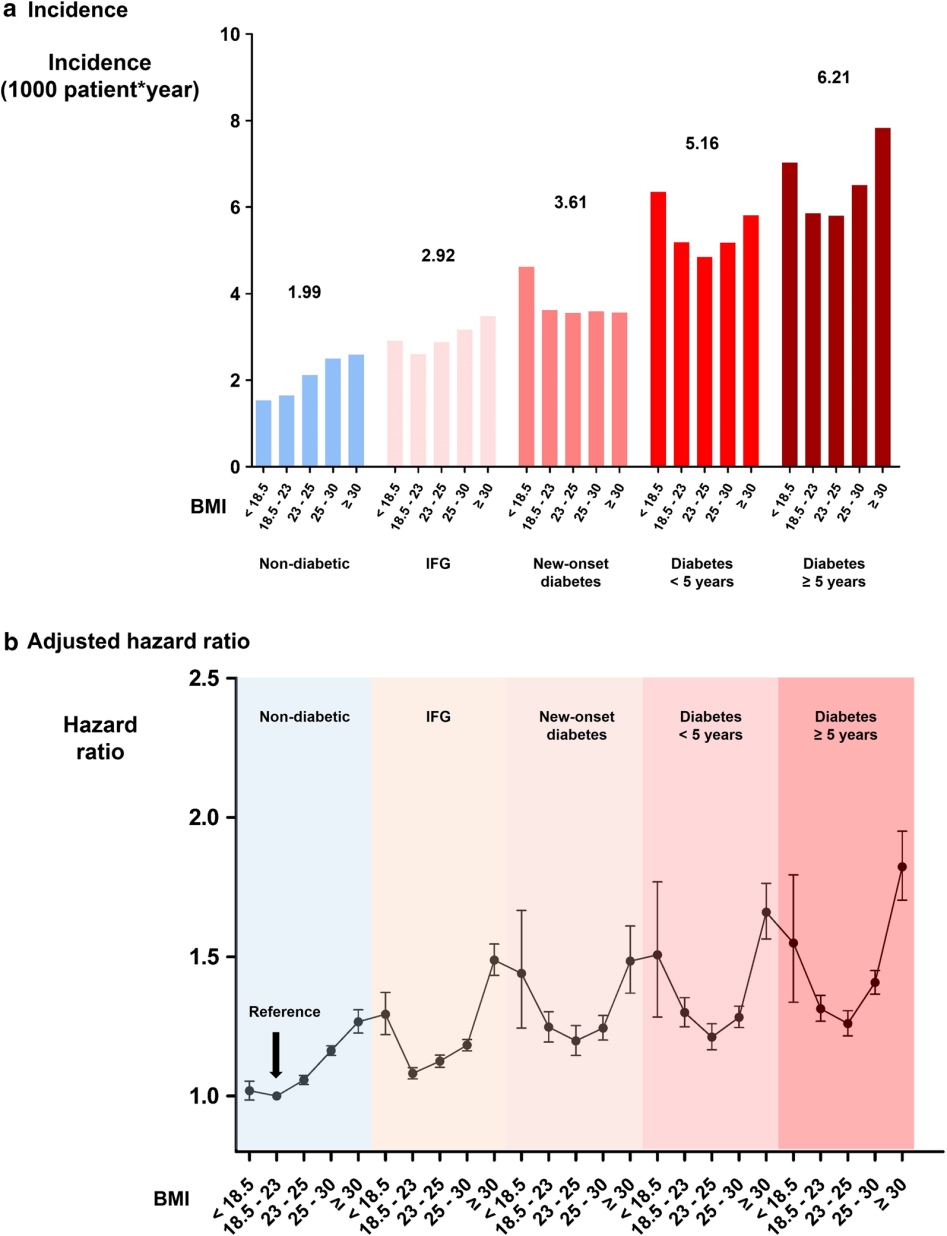
Risk of new-onset AF stratified by diabetic stage and BMI. **a** The incidence of new-onset AF increased significantly according to diabetic stage. The risk also differed significantly within same diabetic stage according to BMI. **b** Adjusted hazard ratio for developing new-onset AF was significantly increased as diabetic stage was aggravated. Obese patients who had diabetes for more than 5 years showed the highest risk. *AF* atrial fibrillation, *BMI* body mass index, *IFG* impaired fasting glucose

Non-diabetic patients with BMI between 18.5 and 23.0 were set as the reference group. There was a gradual increase in the risk of new-onset AF according to diabetic stage (Fig. 2 and Additional file 1: Table S3). Furthermore, patients with IFG also showed a significantly increased risk of new-onset AF compared with non-diabetic patients (incidence = 2.917 vs. 1.994; HR = 1.464; 95% CI 1.449–1.479; p < 0.001; Additional file 1: Table S3). The risk of new-onset AF also differed significantly according to the duration of diabetes (incidence = 3.601, 5.161, 6.206 for new-onset, < 5 years, and ≥ 5 years of diabetes, respectively; Fig. 2 and Additional file 1: Table S3). Being obese showed a synergistic effect with diabetes with obese patients having diabetes for more than 5 years showing the highest risk of new-onset AF (Fig. 2 and Additional file 1: Table S2). In patients without diabetes and IFG, obesity was associated with 23.4% increased risk of new-onset AF (95% CI 1.193–1.276) whereas the risk of new-onset AF was increased by 37.9% (95% CI 1.338–1.421) in patients with diabetes or IFG (p for interaction < 0.001; Additional file 1: Table S4).

### Underweight

The influence of being underweight was analyzed through various subgroup analyses. In patients aged ≥ 65 years, chronic kidney disease, previous stroke, and history of heart disease, being underweight was associated with a significantly increased risk of new-onset AF in non-diabetic patients but not in diabetic patients (Additional file 1: Table S5). For current- or ex-smokers and patients with hypertension, being underweight was associated with a significantly lower risk of new-onset AF in non-diabetic patients (Additional file 1: Table S5).

## Discussion

The major findings of this study are summarized as follows: (1) body weight status and diabetes were both independently associated with the occurrence of new-onset AF; (2) Being underweight, obese or overweight were associated with an increased risk of new-onset AF with obesity showing the highest risk; (3) Not only the presence of diabetes but also its stage was associated with new-onset AF; (4) IFG was also an independent risk factor for development of new-onset AF; (5) A synergistic effect was observed between body weight and diabetes, with obese diabetic patients showing the highest risk of developing AF. This study comprised a large population and evaluated the impact of body weight and diabetes simultaneously on the development of new-onset AF. Due to the very large number of patients, we were able to perform various subgroup analyses including those based on different BMI and diabetes stages.

### Body weight and new-onset AF

Previous studies reported an increased risk of developing AF in overweight and obese patients [20, 22]. The underlying pathophysiology linking being overweight and obese to new-onset AF is not fully understood. Diabetes, hypertension, obstructive sleep apnea, inflammation, increased LA size, and oxidative stress are proposed mechanisms by which being overweight and obese increase the risk of new-onset AF [22]. Our study confirmed that being overweight or obese was a risk factor for new-onset AF with a 12.3% and 32.7% relative increase in the risk, respectively. Being overweight and obese both had significant influence on the development of new-onset AF regardless of the presence of diabetes. We also observed a steep increase in the risk in obese patients suggesting preventing obesity might significantly reduce the risk of new-onset AF. However, avoiding overweight will have more profound impacts on reducing overall AF burden in the Republic of Korea due to a significantly larger number of these patients compared to obese patients. Being overweight is relatively more common in western countries, and therefore, reducing body weight to the normal range will prevent more AF occurrence in these countries. Upper normal body weight status was also associated with the risk of AF development but to a lesser degree: multivariate analysis showed a 4% relative increase in new onset AF risk.

Being underweight is reported to be a risk factor for new-onset AF [17]. Multivariate analysis adjusting for multiple comorbidities and life style in our study also demonstrated that being underweight was associated with an increased risk of new-onset AF. However, underweight patients with no diabetes and IFG did not show an increased risk for new-onset AF compared to the reference BMI group. The association of being underweight and new-onset AF only in patients with diabetes or IFG suggests that insulin resistance may play a role in developing AF in underweight patients. Given that East Asian population has higher prevalence of being underweight compared with western population, to reveal the underlying pathophysiology linking being underweight with new-onset AF and to prevent being underweight will be particularly important in this population. Our study revealed that in smoking patients, being underweight was associated with a low rate of new-onset AF in non-diabetic patients. This protective effect of smoking was not observed in diabetic patients. A similar phenomenon was observed in the study performed by Kang and his colleagues in which being underweight was associated with an increased risk of new-onset AF in non-smokers [17]. The underlying mechanisms for why smoking is associated with a low risk of new-onset AF in non-diabetic underweight patients is an area for future research.

### Diabetes and new-onset AF

Diabetes is an established risk factor for new-onset AF. The Framingham Heart study reported diabetes was an independent risk factor for new-onset AF [23]. A recent study by Huxley et al. reported that diabetes, HbA1c level, and poor glycemic control were associated with an increased risk of AF development [24]. In our study, diabetes was classified into five stages and there was a gradual increase in the risk of new-onset AF as the diabetic stage advanced. The highest incidence of new-onset AF in diabetic patients diagnosed for more than 5 years suggests that the impact of diabetes on AF is cumulative and time dependent. Suggested mechanisms are vascular damage, atrial fibrosis, autonomic dysfunction, and the deleterious effect of advanced glycation end products which are all slow but gradual processes [22, 25–28].

Impaired fasting glucose was not associated with an increased risk for new-onset AF in the previous study [24]. However, a recent study by Lee et al. reported that the risk for new-onset AF was significantly increased in patients with IFG [29]. With a significantly larger sample size, our study showed that IFG is an independent risk factor for new-onset AF (HR = 1.464). Furthermore, in contrast to the study by Lee et al. [29], IFG increased the risk of new-onset AF regardless of BMI in this study. Gradual escalation of the risk for new-onset AF according to diabetic stage is another novel finding of this study.

The impact of diabetes showed a synergistic effect with body weight. Obese patients with diabetes for more than 5 years were at highest risk and therefore, life style modification might be significantly beneficial in these patients. A recent report suggested that oxidative stress and inflammation which exacerbate atrial electrical and structural remodeling are underlying mechanisms linking obesity, diabetes, and AF [30]. Previous reports demonstrated that among patients with established AF, sustained weight loss reduced the burden of AF and symptom severity in a dose-dependent fashion [31, 32]. However, those studies might be limited by sample size and whether weight reduction can also reduce the development of new-onset AF needs further validation. The impact of strict glycemic control on the incidence of new-onset AF is also an interesting area of future research. Among diabetic patients on metformin, dipeptidyl peptidase-4 inhibitor was associated with a lower risk of new-onset AF as compared with other oral hypoglycemic agents suggesting anti-inflammatory effect of dipeptidyl peptidase-4 inhibitor can have additional benefit besides from glycemic control [33, 34].

### Limitations

The current study has several limitations. First, our study is based on an administrative database and is therefore, potentially susceptible to errors arising from coding inaccuracies. Second, we were not able to classify AF type such as paroxysmal and non-paroxysmal. Third, our data is based on East Asian patients and caution is required when applying our results to other ethnic groups. Fourth, we were not able to include several factors which may also affect the incidence of AF, such as drug abuse, prior cardiac surgery, and heart valve disease due to difficulties in identifying these conditions in our nationwide cohort. Finally, we were unable to obtain follow up body weight data which could have addressed an important issue whether body weight loss can reduce new-onset AF.

## Conclusions

Body weight status and diabetes are significantly associated with new-onset AF and both factors act synergistically. Gradual escalation in the risk of new-onset AF was observed with advancing diabetic stage. Body weight and glycemic control will be important for the primary prevention of new-onset AF in a given population.

## Supplementary information

**Additional file 1: Table S1.** Diagnostic codes used in this study. **Table S2.** Impact of body weight stratified by diabetic stage. **Table S3.** Diabetic stage and new-onset atrial fibrillation. **Table S4.** Impact of body weight status and diabetes on new-onset atrial fibrillation. **Table S5.** Influence of underweight on new-onset atrial fibrillation in various subgroups.

## Data Availability

All data generated or analysed during this study are included in this published article and its additional information files.

## Abbreviations

AF: atrial fibrillation
BMI: body mass index
CI: confidence interval
FBS: fasting blood sugar
HR: hazard ratio
ICD-10: International Classification of Disease, Tenth Revision
IFG: impaired fasting glucose
LA: left atrium
NHIS: National Health Insurance Service
RFCA: radiofrequency catheter ablation
WC: waist circumference
