# Supplementary material for: The impact of body weight and diabetes on new-onset atrial fibrillation: a nationwide population based study
Source: Cardiovasc Diabetol. 2019 Oct 1;18:128. doi: 10.1186/s12933-019-0932-z (PMC6774211; doi:10.1186/s12933-019-0932-z)
Supplement: Supplementary file 1 — Additional file 1: Table S1. Diagnostic codes used in this study. Table S2. Impact of body weight stratified by diabetic stage. Table S3. Diabetic stage and new-onset atrial fibrillation. Table S4. Impact of body weight status and diabetes on new-onset atrial fibrillation. Table S5. Influence of underweight on new-onset atrial fibrillation in various subgroups. [file 12933_2019_932_MOESM1_ESM.docx]

**The Impact of Body Weight and Diabetes on New-Onset Atrial Fibrillation: A Nationwide Population Based Study**

Yun Gi Kim, MD,^1^ Kyung-Do Han, PhD,^2^ Jong-Il Choi, MD,^1^* Ki Yung Boo, MD,^1^ Do Young Kim, MD,^1^ Suk-Kyu Oh, MD,^1^ Kwang-No Lee, MD,^1^ Jaemin Shim, MD,^1^ Jin Seok Kim, MD,^1^ Young-Hoon Kim, MD^1^

^1^Division of Cardiology, Korea University College of Medicine and Korea University Anam Hospital, Seoul, Republic of Korea

^2^Department of Biostatistics, College of Medicine, The Catholic University of Korea, Seoul, Republic of Korea

*** Address for correspondence**

Jong-Il Choi, MD, PhD, MHSc

Division of Cardiology, Department of Internal Medicine, Korea University College of Medicine and Korea University Medical Center, Seoul, Republic of Korea

73 Inchon-ro, Seongbuk-gu, Seoul 02841, Republic of Korea.

Tel: 82-2-920-5445

Fax: 82-2-927-1478

E-mail: jongilchoi@korea.ac.kr

The first two authors contributed equally to this work.

**Brief title:** Body weight, diabetes, and atrial fibrillation

**Total word count of the main text:** 2,678

**Table S1.** Diagnostic codes used in this study.

|  | ICD-10 codes |
| --- | --- |
| **Atrial fibrillation** | I48 (2 required for outpatient clinic and 1 for inhospital admission) |
| **Type 2 diabetes** | E11 – E14 |
| **Hypertension** | I10 – I13, I15 |
| **Dyslipidemia** | I78 |
| **Chronic kidney disease** | Based on creatinine checked during national health check-up |
| **Stroke** | I63, I64 |
| **Ischemic heart disease** | I20 – I25 |

**Table S2.** Impact of body weight stratified by diabetic stage.

|  | **Non-diabetic** | **IFG** | **Diabetes** | **Diabetes + IFG** |
| --- | --- | --- | --- | --- |
| **BMI** |  |  |  |  |
| < 18.5 | 1.008 (0.975 – 1.042) | 1.189 (1.120 – 1.262) | 1.119 (1.024 – 1.224) | 1.167 (1.110 – 1.226) |
| 18.5 – 23 | 1 (reference) | 1 (reference) | 1 (reference) | 1 (reference) |
| 23 – 25 | 1.049 (1.033 – 1.065) | 1.033 (1.009 – 1.057) | 0.970 (0.940 – 1.000) | 1.015 (0.996 – 1.034) |
| 25 – 30 | 1.144 (1.127 – 1.161) | 1.095 (1.072 – 1.119) | 1.053 (1.024 – 1.082) | 1.087 (1.068 – 1.106) |
| 30 – | 1.234 (1.193 – 1.276) | 1.368 (1.313 – 1.424) | 1.362(1.301 – 1.425) | 1.379 (1.338 – 1.421) |

Values are adjusted for age, sex, smoking, alcohol consumption, regular physical activity, social income, diabetes, hypertension, and dyslipidemia and expressed as HR (95% CI).

BMI: body mass index; CI: confidence interval; HR: hazard ratio; IFG: impaired fasting glucose; WC: waist circumference.

**Table S3.** Diabetic stage and new-onset atrial fibrillation.

| **Diabetic stage** | **BMI** | **n** | **Event** | **Patient*years** | **Incidence** | **Incidence**  **(subgroup)** | **Model 1 (95% CI)** | **Model 2 (95% CI)** |
| --- | --- | --- | --- | --- | --- | --- | --- | --- |
| **Non-diabetes** | < 18.5 | 311,568 | 3,900 | 2,534,204 | 1.994 | 1.539 | 1.019 (0.986 – 1.053) | 1.033 (0.999 – 1.067) |
|  | 18.5 – 23 | 2,941,493 | 39,803 | 24,194,119 |  | 1.645 | 1 (reference) | 1 (reference) |
|  | 23 – 25 | 1,626,894 | 28,371 | 13,394,103 |  | 2.118 | 1.058 (1.042 – 1.074) | 1.055 (1.039 – 1.072) |
|  | 25 – 30 | 1,680,562 | 34,574 | 13,817,824 |  | 2.502 | 1.163 (1.146 – 1.180) | 1.157 (1.141 – 1.174) |
|  | 30 – | 178,571 | 3,805 | 1,466,237 |  | 2.595 | 1.267 (1.226 – 1.310) | 1.250 (1.209 – 1.293) |
| **IFG** | < 18.5 | 50,373 | 1,155 | 396,945 | 2.917 | 2.910 | 1.294 (1.221 – 1.372) | 1.246 (1.175 – 1.321) |
|  | 18.5 – 23 | 711,589 | 15,030 | 5,781,389 |  | 2.600 | 1.081 (1.061 – 1.102) | 1.077 (1.057 – 1.097) |
|  | 23 – 25 | 579,664 | 13,627 | 4,733,588 |  | 2.879 | 1.125 (1.103 – 1.147) | 1.122 (1.100 – 1.144) |
|  | 25 – 30 | 773,063 | 19,971 | 6,313,235 |  | 3.163 | 1.183 (1.162 – 1.203) | 1.183 (1.163 – 1.204) |
|  | 30 – | 100,793 | 2,858 | 821,259 |  | 3.480 | 1.488 (1.433 – 1.546) | 1.471 (1.416 – 1.528) |
| **New-onset diabetes** | < 18.5 | 5,381 | 181 | 39,166 | 3.607 | 4.621 | 1.440 (1.244 – 1.666) | 1.384 (1.196 – 1.601) |
|  | 18.5 – 23 | 73,854 | 2,118 | 584,604 |  | 3.623 | 1.248 (1.194 – 1.303) | 1.243 (1.190 – 1.298) |
|  | 23 – 25 | 70,803 | 2,028 | 570,136 |  | 3.557 | 1.198 (1.146 – 1.253) | 1.203 (1.150 – 1.258) |
|  | 25 – 30 | 116,752 | 3,388 | 943,145 |  | 3.592 | 1.244 (1.201 – 1.289) | 1.250 (1.207 – 1.295) |
|  | 30 – | 20,627 | 595 | 166,889 |  | 3.565 | 1.484 (1.369 – 1.610) | 1.485 (1.369 – 1.610) |
| **Diabetes < 5 years** | < 18.5 | 3,418 | 150 | 23,611 | 5.161 | 6.353 | 1.507 (1.284 – 1.769) | 1.355 (1.154 – 1.591) |
|  | 18.5 – 23 | 64,437 | 2,598 | 501,429 |  | 5.181 | 1.300 (1.249 – 1.353) | 1.229 (1.181 – 1.279) |
|  | 23 – 25 | 72,824 | 2,819 | 581,162 |  | 4.851 | 1.212 (1.166 – 1.259) | 1.155 (1.111 – 1.200) |
|  | 25 – 30 | 125,559 | 5,225 | 1,008,900 |  | 5.179 | 1.283 (1.246 – 1.322) | 1.219 (1.184 – 1.256) |
|  | 30 – | 23,645 | 1,107 | 190,451 |  | 5.813 | 1.660 (1.564 – 1.763) | 1.553 (1.462 – 1.649) |
| **Diabetes ≥ 5 years** | < 18.5 | 3,761 | 179 | 25,451 | 6.206 | 7.033 | 1.549 (1.337 – 1.794) | 1.401 (1.210 – 1.623) |
|  | 18.5 – 23 | 75,711 | 3,397 | 579,630 |  | 5.861 | 1.313 (1.268 – 1.361) | 1.217 (1.175 – 1.261) |
|  | 23 – 25 | 73,034 | 3,318 | 571,796 |  | 5.803 | 1.260 (1.216 – 1.306) | 1.173 (1.131 – 1.216) |
|  | 25 – 30 | 99,154 | 5,084 | 781,667 |  | 6.504 | 1.408 (1.366 – 1.450) | 1.301 (1.263 – 1.341) |
|  | 30 – | 13,888 | 855 | 109,224 |  | 7.828 | 1.823 (1.703 – 1.951) | 1.645 (1.537 – 1.761) |

Model 1: adjusted for age, sex, smoking, alcohol consumption, regular physical activity, social income, hypertension, and dyslipidemia.

Model 2: adjusted for model 1 plus end stage renal failure, history of heart failure or coronary artery disease, hyperthyroidism, malignancy, and chronic obstructive pulmonary disease.

BMI: body mass index; CI: confidence interval; HR: hazard ratio; IFG: impaired fasting glucose.

**Table S4.** Impact of body weight status and diabetes on new-onset atrial fibrillation.

|  | **BMI** | **n** | **Event** | **Patient*years** | **Incidence**  **(1000 patient*years)** | **HR (95% CI)** | **p for interaction** |
| --- | --- | --- | --- | --- | --- | --- | --- |
| **DM or IFG (-)** | < 18.5 | 311,568 | 3,900 | 2,534,204 | 1.539 | 1.008 (0.975 – 1.043) | < 0.0001 |
|  | 18.5 – 23 | 2,941,493 | 39,803 | 24,194,119 | 1.645 | 1 (reference) |  |
|  | 23 – 25 | 1,626,894 | 28,371 | 13,394,103 | 2.118 | 1.049 (1.033 – 1.065) |  |
|  | 25 – 30 | 1,680,562 | 34,574 | 13,817,824 | 2.502 | 1.144 (1.127 – 1.161) |  |
|  | 30 – | 178,571 | 3,805 | 1,466,237 | 2.595 | 1.234 (1.193 – 1.276) |  |
| **DM or IFG (+)** | < 18.5 | 62,933 | 1,665 | 485,173 | 3.432 | 1.167 (1.110 – 1.226) |  |
|  | 18.5 – 23 | 925,591 | 23,143 | 7,447,051 | 3.108 | 1 (reference) |  |
|  | 23 – 25 | 796,325 | 21,792 | 6,456,681 | 3.375 | 1.015 (0.996 – 1.034) |  |
|  | 25 – 30 | 1,114,528 | 33,668 | 9,046,946 | 3.721 | 1.087 (1.068 – 1.106) |  |
|  | 30 – | 158,953 | 5,415 | 1,287,823 | 4.205 | 1.379 (1.338 – 1.421) |  |

HRs are adjusted for age, sex, smoking, alcohol consumption, regular physical activity, social income, hypertension, and dyslipidemia.

BMI: body mass index; CI: confidence interval; DM: diabetes mellitus; HR: hazard ratio; IFG: impaired fasting glucose.

**Table S5.** Influence of underweight on new-onset atrial fibrillation in various subgroups.

|  |  | **Non-diabetic + IFG** | | **Diabetic** | |
| --- | --- | --- | --- | --- | --- |
|  |  | **HR (95% CI)** | **p for interaction** | **HR (95% CI)** | **p for interaction** |
| Age | < 65 | 0.992 (0.946 – 1.041) | < 0.0001 | 1.029 (0.842 – 1.257) | 0.4692 |
|  | ≥ 65 | 1.121 (1.082 – 1.161) |  | 1.083 (0.982 – 1.194) |  |
| Sex | Male | 0.925 (0.888 – 0.963) | 0.0715 | 1.143 (1.027 – 1.272) | 0.0036 |
|  | Female | 1.013 (0.973 – 1.056) |  | 0.917 (0.787 – 1.069) |  |
| Hypertension | No | 1.091 (1.054 – 1.129) | < 0.0001 | 0.972 (0.850 – 1.112) | 0.3428 |
|  | Yes | 0.885 (0.839 – 0.933) |  | 1.139 (1.014 – 1.279) |  |
| Dyslipidemia | No | 0.996 (0.967 – 1.027) | 0.0054 | 1.123 (1.017 – 1.240) | 0.3129 |
|  | Yes | 1.014 (0.930 – 1.105) |  | 0.961 (0.794 – 1.163) |  |
| Abdominal obesity | No | 1.071 (1.040 – 1.102) | 0.3622 | 1.107 (1.011 – 1.213) | 0.4111 |
|  | Yes | 1.200 (0.999 – 1.443) |  | 1.019 (0.677 – 1.534) |  |
| Smoking | No | 1.109 (1.073 – 1.146) | < 0.0001 | 1.076 (0.968 – 1.197) | 0.6036 |
|  | Yes | 0.634 (0.592 – 0.678) |  | 1.063 (0.909 – 1.243) |  |
| Chronic kidney disease | No | 0.913 (0.886 – 0.941) | < 0.0001 | 1.023 (0.928 – 1.128) | 0.7936 |
|  | Yes | 1.200 (1.101 – 1.307) |  | 1.142 (0.934 – 1.397) |  |
| History of stroke | No | 0.950 (0.916 – 0.985) | 0.0038 | 1.073 (0.970 – 1.186) | 0.5882 |
|  | Yes | 1.139 (0.935 – 1.388) |  | 0.938 (0.562 – 1.566) |  |
| History of angina or MI | No | 0.949 (0.915 – 0.985) | < 0.0001 | 1.099 (0.993 – 1.216) | 0.3170 |
|  | Yes | 1.201 (1.058 – 1.365) |  | 0.818 (0.546 – 1.223) |  |

CI: confidence interval; HR: hazard ratio; MI: myocardial infarction.

Patients with BMI 18.2 – 23 with and without diabetes were set as reference, respectively.
